# Percutaneous balloon-assisted ultrasound-guided direct thrombin embolization of superficial femoral artery pseudoaneurysm: a case series and literature review

**DOI:** 10.1186/s42155-024-00428-8

**Published:** 2024-02-16

**Authors:** Antonio Bruno, Francesco Vendetti, Nicolas Papalexis, Mattia Russo, Dimitris Papadopoulos, Cristina Mosconi

**Affiliations:** Alma Mater Studiorum, Università Di Bologna, Bologna, Italy

**Keywords:** Pseudoaneurysm, Thrombin, Injection, Ultrasound, Balloon, Superficial femoral artery, Ischemia

## Abstract

**Background:**

Superficial femoral artery (SFA) pseudoaneurysms, a rare but potentially life-threatening complication, that can arise after vascular interventions or trauma. This case series explores the efficacy and safety of a minimally invasive treatment modality, percutaneous ultrasound-guided thrombin injection (PUGTI) combined with balloon occlusion, in three patients with SFA pseudoaneurysms.

**Case presentation:**

Three patients (age: 71–82 years; 3 female) with SFA pseudoaneurysms underwent PUGTI with balloon occlusion. The procedure involved direct thrombin injection under ultrasound guidance while occluding the parent artery using a balloon catheter. Follow-up was conducted at 1 week and 1 month post-procedure to assess technical success, complications, and recurrence.

**Conclusion:**

PUGTI combined with balloon occlusion appears to be a safe and effective treatment for SFA pseudoaneurysms, particularly for larger pseudoaneurysms. The procedure is associated with a high technical success rate. Balloon occlusion may offer a safer alternative to direct thrombin injection without occlusion, as it potentially minimizes the risk of complications such as distal thromboembolism.

## Background

Superficial femoral artery (SFA) pseudoaneurysms are infrequent yet serious complications that can develop following traumatic events [1, 2] or they could potentially occur as a complication in as many as 8% of endovascular interventional procedures, well-know as iatrogenic femoral artery pseudoaneurysm (IFAP) [3]. SFA pseudoaneurysms pose a considerable risk of morbidity and mortality, particularly in cases of rupture or resultant distal ischemia [4].

Percutaneous ultrasound-guided thrombin injection (PUGTI) has been reported as a safe and effective treatment for peripheral artery pseudoaneurysms [5, 6]. However, its application in addressing SFA pseudoaneurysms, has been limited,especially in larger ones, or which have a wide or not visible neck on CDUS examination [7]. In this cases direct thrombin injection without occlusion has been linked to an higher rate of complications such as distal thromboembolism [4]. In response to these concerns, PUGTI combined with balloon occlusion has been proposed as a potential alternative treatment modality for SFA pseudoaneurysms.

This case report presents the outcomes of three patients with SFA pseudoaneurysms who underwent PUGTI combined with balloon occlusion as a minimally invasive treatment option.

The objective of this work is to contribute,describing three cases, to the growing body of evidence supporting the use of PUGTI with balloon occlusion as a viable treatment for SFA pseudoaneurysms, particularly in the context of larger pseudoaneurysms [6] with wide or not visible neck on CDUS.

## Case report

We describe three cases of Patients presenting pseudoaneurysm of the right superficial femoral artery treated by percutaneous injection of thrombin under combined fluoroscopic and ultrasound guidance.

The first case concerns a 76 -year-old patient who was transferred to our hospital due to a painful swelling at the right thigh for 12 h after an episode of, sepsis-induced Multi-Organ Dysfunction (MODS). On clinical examination, swelling of soft tissue at the right thigh was found, without sign of right lower extremity ischemia.

Color Doppler Ultrasonography (CDUS) was performed confirming a poly lobulated pseudoaneurysm of the superficial femoral artery, just distal to the femoral bifurcation, measuring 6 × 23 mm, with tight neck and with a large hematoma extended into adjacent soft tissues.

The patient was referred to the Interventional Radiology unit for the percutaneous, ultrasound-guided thrombin injection. Before the procedure Computed tomography with contrast medium of the lower extremities was performed, to more accurately asses the pseudoaneurysm sac and the relation between its neck and the parental artery to reduce the risk of thrombin leakage to the arterial system.

The patient underwent diagnostic angiography from the left common femoral artery approach through a 5.0-French sheath (Terumo, Tokyo, Japan). A pseudoaneurysm of the right superficial femoral artery, measuring 6 × 23 mm, composed of two connected sacs and with a tight neck was presented. Ultrasound-guided puncture of both pseudoaneurysm’s sacs, at different times, under local anesthesia was carried out with a needle 18G. We inflated an EverCross 0.035″ (PTA Balloon Catheter Medtronic AVE, Santa Rosa, CA, USA) of 4 × 60 mm in front of the pseudoaneurysm’s neck to block blow into the pseudoaneurysm. 3 ml of thrombin was injected (Floseal hemostatic matrix 5 ml, Baxter, Deerfield, Illinois, USA.) under combined fluoroscopic and ultrasound guidance until the sac is totally filled. The balloon catheter was deflated 3 min after the injection was finished. On the control angiography, a total exclusion of pseudoaneurysm was obtained. The examination at 1-month postoperative was unremarkable and no complication nor discomfort was found.

**Fig. 1:**
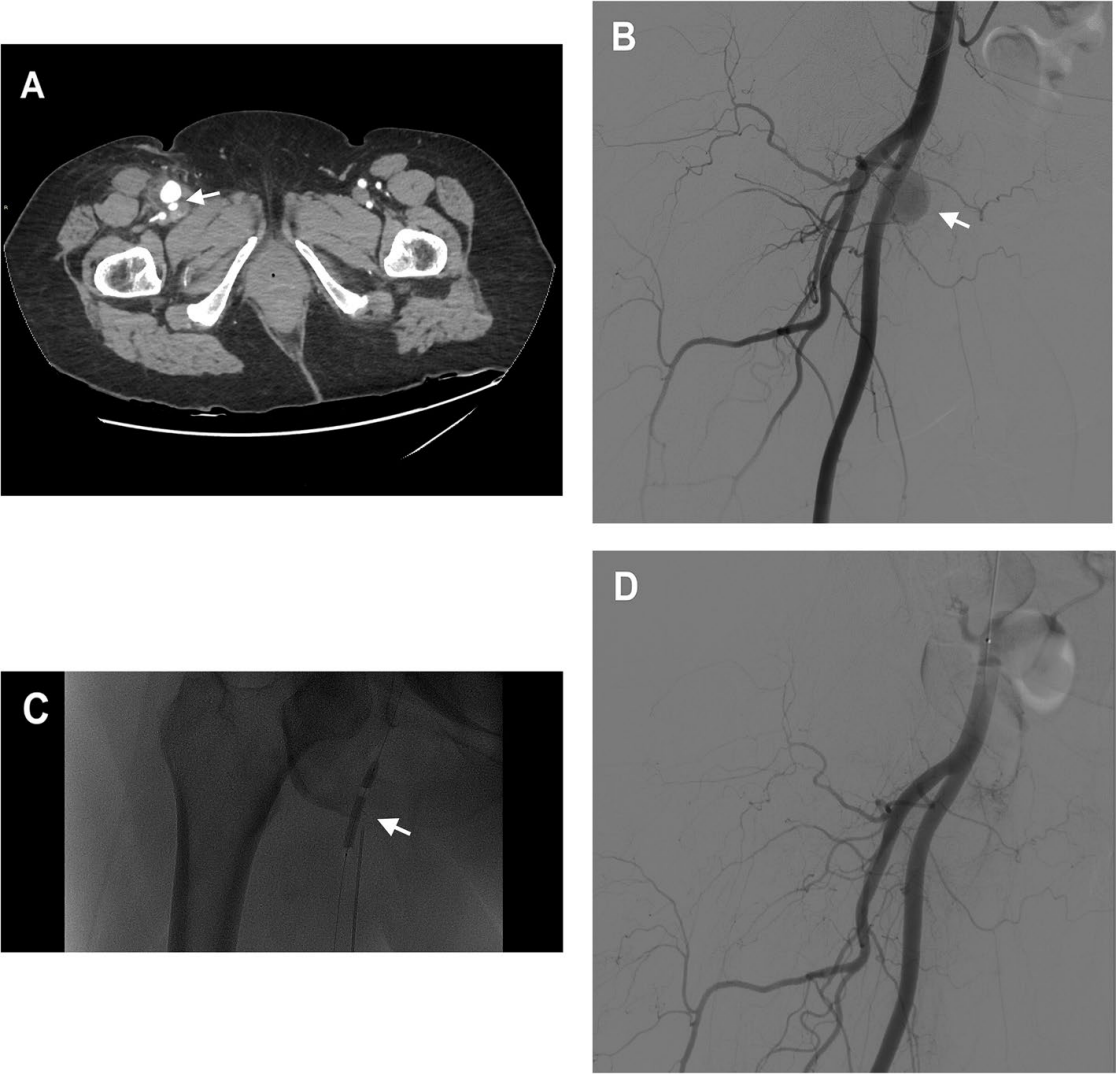
A 71 years-old patient who suffered from a post-catheterization pseudoaneurysm of the right SFA with a short and wide neck, measuring 30 × 26 mm. **a** Contrast-enhanced CT scan in the arterial phase showing the SFA pseudoaneurysm (arrow) **b** Digital subtraction angiography (DSA) of the common femoral artery using contralateral femoral access showing the large pseudoaneurysm of the SFA (arrow) **c** procedural DSA showing balloon occlusion of the neck of the pseudoaneurysm while performing percutaneous thrombin injection (arrow) and **d** post-procedural DSA showing absence of contrast uptake by the pseudoaneurysmal sack with normal opacization of the CFA

The same technique was performed for a 71 and an 80 -year-old patients,who underwent to endovascular treatment of acute Stroke. In both cases the follow-up cranial CT with contrast, complemented by angiographic scans of the lower limbs basing on clinical suspicion, was performed and a pseudoaneurysms of SFA was reported. These were both composed by a single sac and a wide neck, the first measuring 30 × 26 mm, the other measuring 27 × 21 mm (Fig. 1).

As well as the previous case the patients underwent diagnostic angiography and the pseudoaneurysms of the right superficial femoral artery were confirmed.

Using the same approach an ultrasound-guided puncture of the pseudoaneurysm sac under local anesthesia was carried out with a needle 18G x15cm and Advance (18LP 0.018″) was inflated (PTA Balloon Catheter, Cook medical, Bloomington, Indiana, USA.) of 4 × 60 mm in front of the pseudoaneurysm’s neck. 1,5 ml of thrombin was injected (Floseal hemostatic matrix 5 ml, Baxter, Deerfield, Illinois, USA) As in the first case the control angiography at 1-week after reveled a total exclusion of pseudoaneurysm.

## Conclusion

Superficial femoral artery pseudoaneurysm occurs as a complication in as many as 8% of endovascular interventional procedures. In particular when arterial wall defect on the puncture site fails to seal, causing a pulsatile hematoma formation communicating with the arterial lumen, which is confined by surrounding tissues [6].

IFAP usually tent to thrombose spontaneously over the time [8] but when they persist there are several treatment options to be considered. UGCT, based on applying pressure using the ultrasound transducer at the neck of the pseudoaneurysm until the flow through it is stopped for approximately 10–20 min, it is a safe and effective method [9, 10] but is more suitable for non obese patients without coagulation disorders, while it is painful and taking a long time [11–13].

Traditional surgical repair has been the primary management strategy for SFA pseudoaneurysms; however, this approach is associated with substantial risks such as infection, bleeding, and damage to adjacent structures [14]. In the same way the covered stent is considered a highly effective and safe method, but it requires anticoagulant therapy for a prolonged period and is sometimes not available in the required size [11]. In order to avoid these discomforts for the patient, it was decided to discard this option.

As endovascular strategies of treatment we have some options such as: the placement of embolization coils from a percutaneous access, but in many cases the coils would not guarantee complete filling of the pseudoaneurysm with persistent flow between them [15]. Another option is glue [16], which, however, is difficult to administer safely without experience and may have undesirable long-term effects. In addition, if part of the drug leaks through the collar, it could cause necrosis or infarction [17]. For these reasons, those choices were ruled out.

For thrombin the matter is entirely different, being a physiological coagulant agent that is readily broken down by the body and it is very effective in aiding the coagulation of static blood within pseudoaneurysms and yet there is little chance that it would affect distal vascular districts if a small amount of it escaped downstream [18–20]. Direct percutaneous injection of thrombin has already been used in some centers with a high success rate [10, 17, 21, 22], regardless of the coagulation status and size of the pseudoaneurysm [21, 23]. The major risk of the procedure, thrombin being a liquid embolic agent, is limb ischemia due to thrombin escaping through the neck of the pseudoaneurysm, possibly resulting in limb ischemia [13, 24]. This, in particular, is due to the characteristically circular flow within the pseudoaneurysm, with rapid outflow within the artery in the diastolic phase determining the thromboembolic risk [15, 25]. However, to circumvent these thrombo-embolic risks with certainty and safety, a number of precautionary techniques have been described in the literature [10, 26–28].

In our cases we propose a method to occlude pseudoaneurysm with a wide neck or with a tight neck not clearly visualized in the diagnostic phase, placing a catheter with an occlusion balloon, inflated at low pressure, in front of in the artery of origin. With this technique, there is little chance of blood flow into the lumen of the pseudoaneurysm due to the blockage of the collar [15, 29]. This procedure also helps to overcome the disadvantages of previous methods such as poor coagulation status, obesity, a large hematoma or the lack of special resolution about the pseudoaneurysms tight neck on the CDUS examination [7]. However, the balloon-assisted method may have certain disadvantages. Firstly, there is the risk of a persistent refilling of the pseudoaneurysm due to insufficient thrombin supply. This can however be resolved by repeating the procedure until the bag is completely filled. Secondly, this technique requires contralateral femoral access which obviously carries the risk of incurring the same complication [8].

## Data Availability

All the data used for writing this manuscript were taken from the medical records of the patients treated with percutaneous ultrasound-guided thrombin injection (PUGTI) combined with balloon occlusion. The figures used in this paper where taken from the PACS system.

## Abbreviations

SFA: Superficial femoral artery
IFAP: Iatrogenic femoral artery pseudoaneurysm
CDUS: Color Doppler Ultrasonography
PUGTI: Percutaneous ultrasound-guided thrombin injection
MODS: Multi-Organ Dysfunction
UGCR: Ultrasound-guided compression
